# Luteolin Nanomedicine with Stimulus-Driven Traceless Release for Targeting Treatment of Atherosclerosis by Enhancing Lipid Efflux

**DOI:** 10.34133/research.0754

**Published:** 2025-07-11

**Authors:** Songzan Chen, Zhaojing Wang, Zhida Shen, Di He, Lijuan Liu, Lingbo Qian, Boxuan Ma, He Huang

**Affiliations:** ^1^ Department of Cardiology, Sir Run Run Shaw Hospital, School of Medicine, Zhejiang University, Hangzhou, China.; ^2^ Zhejiang Key Laboratory of Cardiovascular Intervention and Precision Medicine, Hangzhou, China.; ^3^ Engineering Research Center for Cardiovascular Innovative Devices of Zhejiang Province, Hangzhou, China.; ^4^ School of Basic Medical Sciences & Forensic Medicine, Hangzhou Medical College, Hangzhou, China.

## Abstract

Atherosclerotic plaques pose a substantial risk for life-threatening cardiovascular complications due to their propensity to trigger acute clinical events. Foam cell formation, resulting from dysregulated lipid homeostasis, serves as a pivotal pathological hallmark in the progression of atherosclerosis. In this study, we presented a precision therapeutic strategy targeting foam cells of multiple origins. The carrier-free nanomedicine Dlut based on luteolin was constructed, featuring CD44 active targeting and stimulus-driven traceless release. Dlut demonstrated active foam cell targeting capability and complete disintegration triggered by oxidative stress and acidic microenvironment, enabling a traceless release of luteolin. Transcriptomic profiling revealed that Dlut inhibited foam cell formation by accelerating lipid efflux. In vivo studies further demonstrated that Dlut significantly reduced plaque burden and improved plaque stability, highlighting a translational potential of atherosclerosis.

## Introduction

Atherosclerosis is a systemic disease characterized by chronic vascular inflammation and degeneration, although its exact mechanisms remain largely undefined [1]. Excessive cholesterol accumulation and subsequent formation of foam cells are well-established pathological hallmarks [2]. Foam cells originate from both immune cells and nonimmune cells [3]. Among these cell types, smooth muscle cells (SMCs) and macrophages served as the main source of foam cells [4]. Therapeutic targeting strategies that delay foam cell formation or alleviate intracellular lipid burden have been proven effective for atherosclerosis in animal models and clinical fields [5–7].

Recently, strategies based on natural products have shown great potential for the early prevention and treatment of atherosclerosis [8]. A wide range of natural compounds have exhibited excellent anti-atherosclerotic properties [9]. Especially, plant-derived natural products, such as flavonoids, which include flavone, flavanone, isoflavone, and dihydrochalcone, have shown great treatment potentials [10].

Luteolin (Lut), a naturally occurring flavone found in fruits and vegetables, is known for its various biological and pharmacological properties [11,12]. Previous studies have demonstrated that Lut possesses a variety of biological and pharmacological activities [13,14]. Meanwhile, Lut exhibited promising vascular protective potential [15,16]. As for atherosclerosis, Lut possesses therapeutic value for its regulatory effects under inflammation [17]. However, the therapeutic application of Lut for vascular diseases is limited by high lipophilicity and poor oral bioavailability [18]. Furthermore, emerging evidence suggested that Lut may exert cytotoxic effects under specific conditions, raising concerns about its potential adverse effects in therapeutic applications [19]. These limitations challenge the efficiency and translational application, underscoring the need for an effective drug delivery strategy. To effectively inhibit foam formation and suppress the progression of atherosclerosis, a foam cell targeting strategy with Lut might help overcome these problems.

For this strategy to succeed, it is critical to have a candidate cell membrane receptor that is expressed in abundance in foam cells and shows a disease-specific responsive expression [20]. Emerging evidence highlights CD44, a type I transmembrane glycoprotein, as a pivotal therapeutic target for modulating atherosclerosis progress within plaques [21,22]. CD44 is broadly expressed in its standard isoform across diverse cellular populations and becomes activated under pathological conditions on endothelial cells, SMCs, and immune cells [23,24]. It also orchestrates the initiation of plaque development through multiple mechanisms [25]. However, the function of CD44 during foam cell formation remains largely unknown. Recent studies have implicated CD44 in the formation of macrophage-derived foam cells via CD36 up-regulation [26,27]. However, the role of CD44 expression in SMC-derived foam cells remains unclear, making a targeting strategy to inhibit dual-lineage-derived foam cell formation within plaques a persistent challenge.

The evolution of nanotherapeutic platforms over the past decade has catalyzed the clinical translation of multifunctional systems for managing atherosclerosis and other diseases [28–31]. The pathologically disrupted endothelial architecture in atherosclerotic plaques creates a permissive microenvironment for nanoparticle accumulation [32,33]. By tailoring the nanoparticles to the distinct pathological hallmarks of atherosclerotic plaques, the nanoparticles can be engineered for active targeting of these lesions while significantly reducing immune recognition and subsequent clearance [34–36]. Collectively, the optimized nanoplatform minimizes off-target effects associated with nonspecific drug distribution while enhancing site-specific accumulation, thereby offering a promising strategy for improved theranostic efficacy [37,38].

**Fig. 1.**
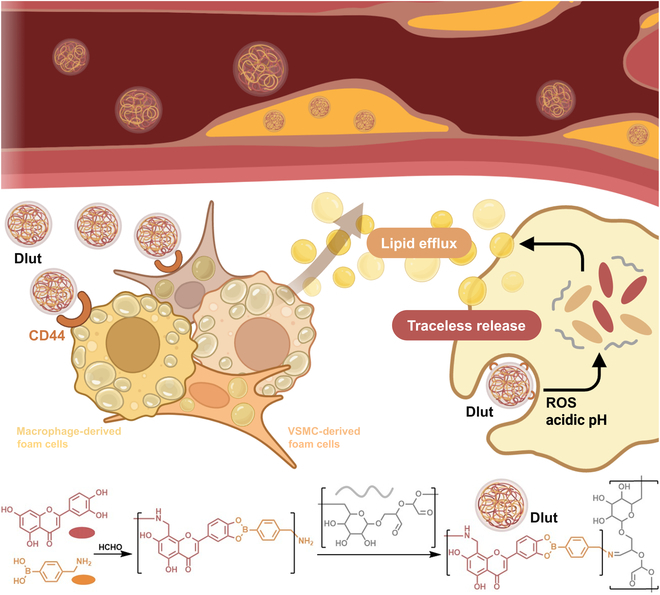
Illustration of the construction of carrier-free Dlut nanomedicine with stimulus-driven traceless release and CD44-targeting ability, which treated atherosclerosis by enhancing lipid efflux.

In this study, we developed a targeted nanomedicine Dlut, based on Lut for the dual lineage foam cell targeting therapy within atherosclerotic plaques. The up-regulation of CD44 in foam cell induction in vitro derived from both SMC and macrophages was verified. Lut was aggregated into carrier-free nanoparticles using 4-(aminomethyl) phenyl boronic acid, and the nanomedicine was grafted on the surface with the oxidized dextran (oxDEX) [39,40]. The oxDEX-functionalized surface enables Dlut nanomedicine to retain structural stability in the bloodstream post-injection and facilitates their binding to overexpressed CD44 receptors on foam cells through the injured endothelium [41,42]. Upon reaching the atherosclerotic lesion site, the Dlut nanomedicine responded to the local oxidative stress and acidic microenvironment in atherosclerotic lesions [43,44]. This triggered a complete disintegration of Dlut nanoparticles, resulting in the traceless release of Lut. Transcriptome sequencing analysis elucidated that the underlying regulatory roles of Dlut during foam cell formation inhibited atherosclerotic progression primarily by activating the pathway of accelerated lipid efflux (Fig. 1). Our findings suggested that Dlut nanomedicine enables efficient Lut accumulation in foam cells derived from dual lineages, offering a pioneering therapeutic candidate for atherosclerosis.

## Results

### Preparation and the stimuli responsiveness of Dlut nanomedicine

**Fig. 2.**
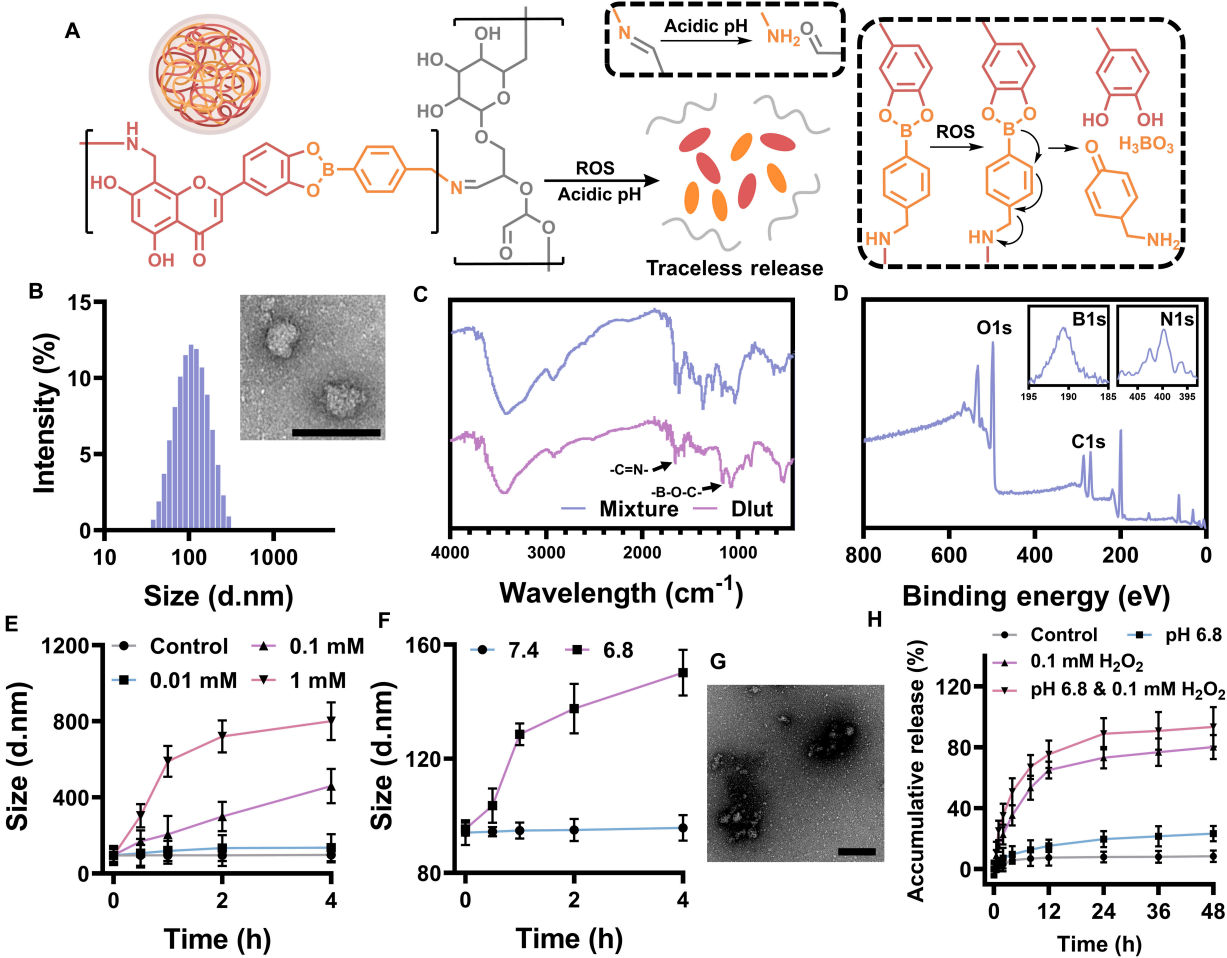
Characterization and the stimuli responsiveness of Dlut nanomedicine. (A) Chemical structure and the illustration of Dlut disintegrating by acidic pH and ROS. (B) Distribution of particle size and the TEM image belonging to Dlut. (C) FTIR spectrum of Dlut nanomedicine and the mixture of Lut, 4-(aminomethyl) phenyl boronic acid, and oxDEX. (D) XPS spectrum of Dlut. (E) Size variation of Dlut under different concentrations of H_2_O_2_. (F) Differentiation of Dlut size under various pH. (G) TEM image of Dlut in H_2_O_2_ (0.1 mM) at pH 6.8 after 6 h of incubation. (H) Accumulative release of Lut from nanomedicine when under various conditions. All the scale bars were 200 nm. Data in (E), (F), and (H) are expressed as mean ± SD (*n* = 3).

The Dlut nanomedicine was prepared through a combination of borate ester reaction, phenolic condensation, and Schiff’s base reaction, which also endowed Dlut with the sensibility to reactive oxygen species (ROS) and acidic pH (Fig. 2A). Dlut was constructed with a diameter of 89.46 nm (polydispersity index 0.109), and a spherical morphology of the nanomedicine was also discovered under a transmission electron microscope (TEM) (Fig. 2B). The chemical bonding in Dlut was also characterized to indicate the formation of nanomedicine. According to the spectrum of x-ray photoelectron spectroscopy (XPS) and Fourier transform infrared (FTIR) (Fig. 2C and D), the elemental peak of C, O, N, and B was found, while the imine linkage and boric acid ester were observed to form with the characteristic absorption bands at 1,150 and 1,645 cm^−1^.

Furthermore, the responsiveness of Dlut nanomedicine under ROS and acidic pH was evaluated by measuring the variation in particle size. When the H_2_O_2_ concentration was greater than 0.1 mM, the particle size of Dlut increased significantly (Fig. 2E), and the nanostructure could also be interrupted by an acidic environment at pH 6.8 (Fig. 2F). Notably, Dlut demonstrated a more pronounced disintegration under ROS conditions. In addition, the disassembly of Dlut under ROS, as well as acidic pH, was also confirmed by TEM (Fig. 2G), which revealed a clear decomposition of nanoparticles and the presence of irregular fragments.

Moreover, the traceless release of Lut was further investigated by measuring the amount of free Lut after testing the nanomedicine under various conditions. As shown in Fig. 2H, while Dlut was interrupted by acidic pH to some extent with only 23% of the payload released after 48 h, the ROS condition could trigger a stronger release of Lut from the disintegration of Dlut, with nearly 80% released after 48 h. Together, the Dlut nanomedicine indicated a rapid sensitivity when under acidic pH along with oxidative stress, where about 80% of Lut was delivered in 12 h and nearly 90% was released after 48 h.

### In vitro internalization and treatment on foam cells

**Fig. 3.**
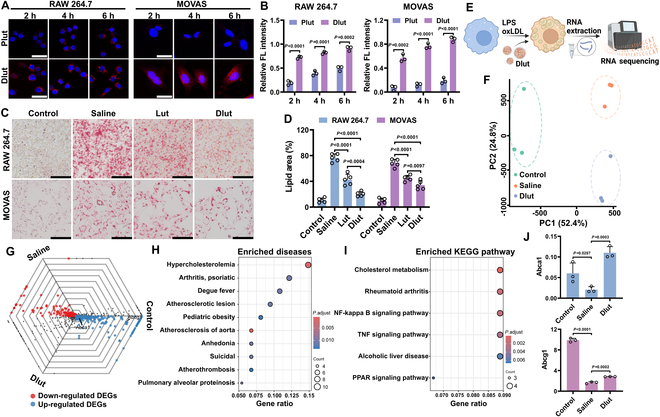
In vitro therapeutic ability of Dlut. Fluorescent (A) and normalized intensity (B) of foam cells derived from RAW 264.7 and MOVAS cells treated with Plut and Dlut for the indicated time. Scale bars, 50 μm. ORO staining (C) and quantification (D) of foam cells with different stimulations. Scale bars, 100 μm. (E) Schematic representation of the design for in vitro experiment on foam cells treated with Dlut and subsequent RNA sequencing analysis. (F) PCA plot of 9 samples in foam cells with various treatments based on the genes. (G) Illustration of genes in foam cells under different stimulations by the triwise plot. (H) Top 10 significant DisGeNET enrichment items analyzed based on DEGs. (I) Kyoto Encyclopedia of Genes and Genomes (KEGG) pathway enrichment analysis of identified DEGs. (J) Fragments per kilobase of transcript per million mapped reads (FPKM) normalized expression of Abca1 and Abcg1 according to the sequencing results. Data in (B), (D), and (J) are expressed as mean ± SD (*n* = 3).

Given the diverse origins of foam cells and unique features of foam cells, 2 main cell types, macrophages (RAW 264.7) and SMCs (MOVAS), were utilized for the induction of foam cells in vitro with the activation of lipopolysaccharide (LPS) and oxidized low-density lipoprotein (oxLDL). CD44 was found to be significantly up-regulated in both cell types and polarization phenotype (Figs. S1 and S2). Therefore, to enable selective targeting, the nanoparticles were engineered to interact with CD44. First, the localized therapeutic potential of Dlut targeting lipid-laden foam cells was investigated in vitro. Fluorescence imaging was used to trace the internalization of Dlut in foam cells derived from macrophages and SMC (Fig. 3A). Moreover, the nanoparticles without CD44 selection (Plut) were used as the negative control. We found increasing fluorescence intensity observed in cells over a 6-h period, which suggested a progressive accumulation of Dlut nanoparticles (Fig. 3B). What is more, the extra hyaluronic acid (HA) was added to block CD44 for indicating the specific uptake. As shown in Fig. S3, a concentration-dependent blockage of CD44 by HA could inhibit the internalization of Dlut, which indicated that the specific endocytosis of Dlut was mediated by CD44.

Atherosclerosis is characterized by chronic inflammation and the accumulation of lipids, processes primarily driven by the involvement of macrophages and foam cells. Hence, based on the previous studies on the anti-inflammatory and antioxidant properties of Lut, Dlut was expected to achieve the inhibition of foam cell formation. Accordingly, the addition of Dlut was found to significantly inhibit foam cell formation in both macrophages and SMC (Fig. 3C). Compared to free Lut, Dlut exhibited greater efficiency in preventing foam cell formation (Fig. 3D). Furthermore, the nanomedicine without stimuli responsiveness (NRlut) and 2 typical agents (dexamethasone and atorvastatin) were also used to investigate the therapeutic outcome of Dlut (Fig. S4). NRlut exhibited diminished therapeutic efficacy in the absence of stimuli responsiveness, while Dlut indicated a similar therapeutic effect with dexamethasone, and even showed a better outcome than atorvastatin in treating foam cells originating from MOVAS. These results highlight Dlut’s ability to suppress foam cells of dual cellular origin in vitro, supporting its potential as an effective therapeutic agent for in vivo treatment of atherosclerosis.

### Molecular mechanism studies of Dlut by the transcriptome analysis

According to in vitro demonstration in foam cells from macrophages and SMC, Dlut exhibited considerable anti-atherosclerosis activity. Previous studies have demonstrated that Lut possesses strong antioxidant properties applicable to various diseases [45–47]. Nevertheless, the molecular mechanism of Dlut nanomedicine modulating inflammation and lipid metabolism remains unclear. Thereafter, to further the mechanism of Dlut regulating foam cells, transcriptome analysis was further investigated. As shown in Fig. 3E, whole transcriptome RNA sequencing was performed on foam cells following treatment with Dlut. First, principal components analysis (PCA) was performed on all 9 samples (Fig. 3F). Significant heterogeneity was found among the 3 groups, whereas relatively excellent homogeneity existed within the samples per group. Given the distinct properties among the 3 groups, the triwise plot was illustrated to identify potential hub genes (Fig. 3G). We primarily focused on the genes reversely regulated by Dlut under oxLDL stimulation (red and blue dots).

A total of 70 Dlut up-regulating differentially expressed genes (DEGs) and 37 Dlut down-regulating DEGs were identified (Fig. S5). Interestingly, 2 genes, Abca1 and Abcg1, which played important roles in lipid efflux, were identified (Fig. 3G). The 107 Dlut regulated genes were mainly enriched in pathways related to the pathogenesis of hypercholesterolemia, atherosclerotic lesions, atherosclerosis of the aorta, and atherothrombosis (Fig. 3H). Moreover, we found a strong association between these genes and the pathways of cholesterol metabolism, indicating that lipid metabolism might play an important role in the Dlut regulated foam cell formation (Fig. 3I) [48,49]. In addition, the up-regulated expression of Abca1 and Abcg1 was also confirmed (Fig. 3J).

### Dlut alleviated lipid burden by accelerating lipid efflux

**Fig. 4.**
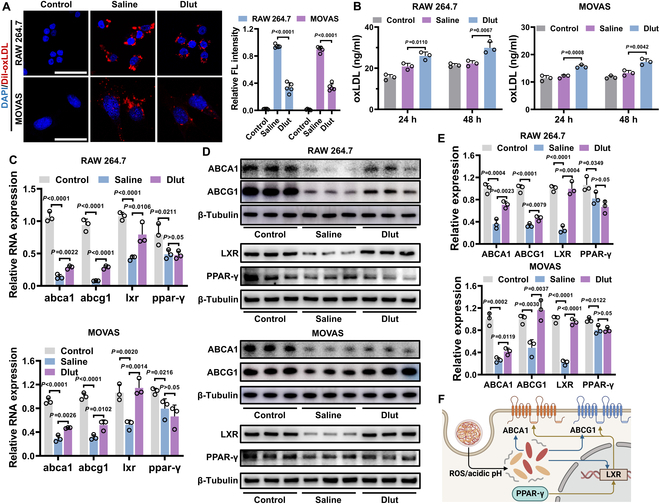
Dlut alleviated foam cell formation by enhancing lipid efflux. (A) Less residual Dil-oxLDL visualized under Dlut stimulations by enhancing lipid efflux (left) and normalized intensity statistics (right). Scale bars, 50 μm. (B) Significantly increased oxLDL content in the supernatant medium in both cells treated with Dlut. (C) Dlut significantly up-regulated the mRNA expression of LXR, ABCA1, and ABCG1, while no significant change of PPARγ mRNA was detected. (D) Dlut increased the protein expression of LXR, ABCA1, and ABCG1, while no obvious activation of PPARγ was found. (E) Normalized expression of selected proteins in different groups. (F) Illustration of the mechanism of Dlut for treatment of foam cells. Data in (A) to (C) and (E) are expressed as mean ± SD [(A): *n* = 5; (B), (C), and (E): *n* = 3].

To validate the up-regulation of ABCA1 and ABCG1 by sequencing technologies, we investigated the lipid content within foam cells after 4 h of incubation by Dil-oxLDL. Consistent with earlier findings, reduced oxLDL accumulation was observed in foam cells derived from both macrophages and SMCs, indicating enhanced efflux activities in these cells (Fig. 4A). Accordingly, oxLDL levels in the supernatant were significantly increased in Dlut-treated cells (Fig. 4B). In addition, the up-regulated mRNA expression of ABCA1 and ABCG1 was validated by quantitative polymerase chain reaction (qPCR) (Fig. 4C). Previous studies reported that ABCA1 and ABCG1 were regulated by the peroxisome proliferator-activated receptor-liver X receptor (PPAR-LXR) pathway [50]. To explore this mechanism, the expression levels of PPARγ and LXR were also examined. The expression of LXR mRNA was significantly enhanced by Dlut, whereas no significant change in PPARγ mRNA expression was found, suggesting the involvement of posttranslational mechanisms. These findings were further confirmed at the protein level, with statistically significant differences observed in both cell types (Fig. 4D and E and Fig. S6). To further investigate the mechanism of Dlut influencing LXR-ABCA1/ABCG1, the inhibitor was added to block the LXR and ATP-binding cassette transporter (ABC) pathway. As shown in Fig. S7, after being treated with the LXR inhibitor [glycogen synthase kinase (GSK)], the up-regulation of LXR, ABCA1, and ABCG1 was counteracted. On the other hand, when the ABC inhibitor (DIDS) was added, the up-regulation of ABCA1 and ABCG1 by Dlut was weakened, while the promotion to LXR was maintained. The results showed a direct regulation of Dlut to LXR, ABCA1, and ABCG1, which synergistically accelerated the lipid efflux from foam cells (Fig. 4F).

### In vivo therapy against atherosclerosis

Under clinical circumstances, achieving effective medication dosage within atherosclerotic plaques is critical for therapeutic success. To evaluate the in vivo efficacy of Dlut, the nanomedicine was administered using animal models. Considering the poor water solubility of Lut and the potential toxicity of long-term dimethylsulfoxide (DMSO) injection, a control group was established using nontargeted nanoparticles (Plut), which lacked plaque-targeting capability.

**Fig. 5.**
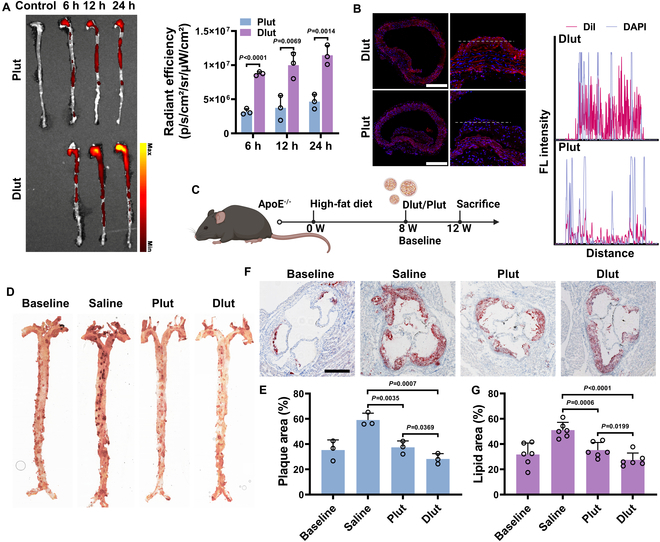
In vivo efficient accumulation and treatment of Dlut in the atherosclerotic lesions. (A) Ex vivo fluorescence imaging and quantitative analysis of Plut and Dlut accumulation in aortic tissues at various time points. (B) Fluorescence microscopy images showing the spatial distribution of nanomedicine in aortic root sections, accompanied by representative fluorescence emission spectra. Scale bars, 500 μm. (C) Schematic representation of the therapeutic protocol for atherosclerosis. Photographs (D) and quantitative analysis of relative plaque area (E) on the en face of ORO-stained aortas. Section of ORO-stained aortic roots (F) and statistical analysis (G). Quantitative data presented in (A), (E), and (G) are expressed as mean ± SD [(A) and (E): *n* = 3; (G): *n* = 6].

The preliminary in vivo biosafety evaluation and pharmacokinetics of Dlut were initially conducted in ApoE^−/−^ mice and revealed no evidence of acute toxicity in major organ systems (Fig. S8) and a satisfactory circulation time (Fig. S9). Subsequently, the active targeting capability of Dlut mediated via its oxDEX surface was compared to Plut, which featured a nontargeted polyethylene glycol (PEG) coating (Fig. 5A). There was a higher accumulation of Dlut found in the lesions of diseased aortas (Fig. 5B), and the immunofluorescence suggests that Dlut was colocalized with macrophages and SMCs (Fig. S10). Leveraging a passive targeting mechanism facilitated by the endothelium disruption, Plut exhibited significant aortic accumulation within 24 h post-injection. In contrast, Dlut nanoparticles functionalized with oxDEX demonstrated superior targeting efficiency to atherosclerotic plaques through CD44-mediated active targeting mechanisms. Additionally, Plut exhibited greater accumulation in the liver and kidney compared to Dlut, suggesting enhanced metabolic processing of Plut relative to Dlut (Fig. S11).

Next, we evaluated the in vivo therapeutic efficacy of Dlut in atherosclerotic animal models. As outlined in the experimental timeline outlined in Fig. 5C, mice were euthanized at 12 weeks, and atherosclerotic plaque burden in the aorta was initially assessed via Oil red O (ORO) staining (Fig. 5D). Compared to the saline-treated group, Plut demonstrated a significant reduction of ORO-positive areas in the aortic tissues, indicating an attenuation of atherosclerotic plaque progression. This underscores the therapeutic efficacy of Lut-based therapy even in the absence of active targeting mechanisms.

Importantly, Dlut nanoparticles, equipped with oxDEX-mediated active targeting, demonstrated superior anti-atherosclerotic efficacy compared to Plut, with a markedly greater reduction in ORO-positive plaques (Fig. 5E). To further investigate treatment effects, aortic root sections were subjected to multivariate analysis (Fig. 5F). Consistent with the en face aortic ORO staining, the analysis of aortic root sections confirmed that Dlut exhibited the strongest inhibitory effect on plaque progression, as reflected by the smallest lipid deposition area (Fig. 5G).

**Fig. 6.**
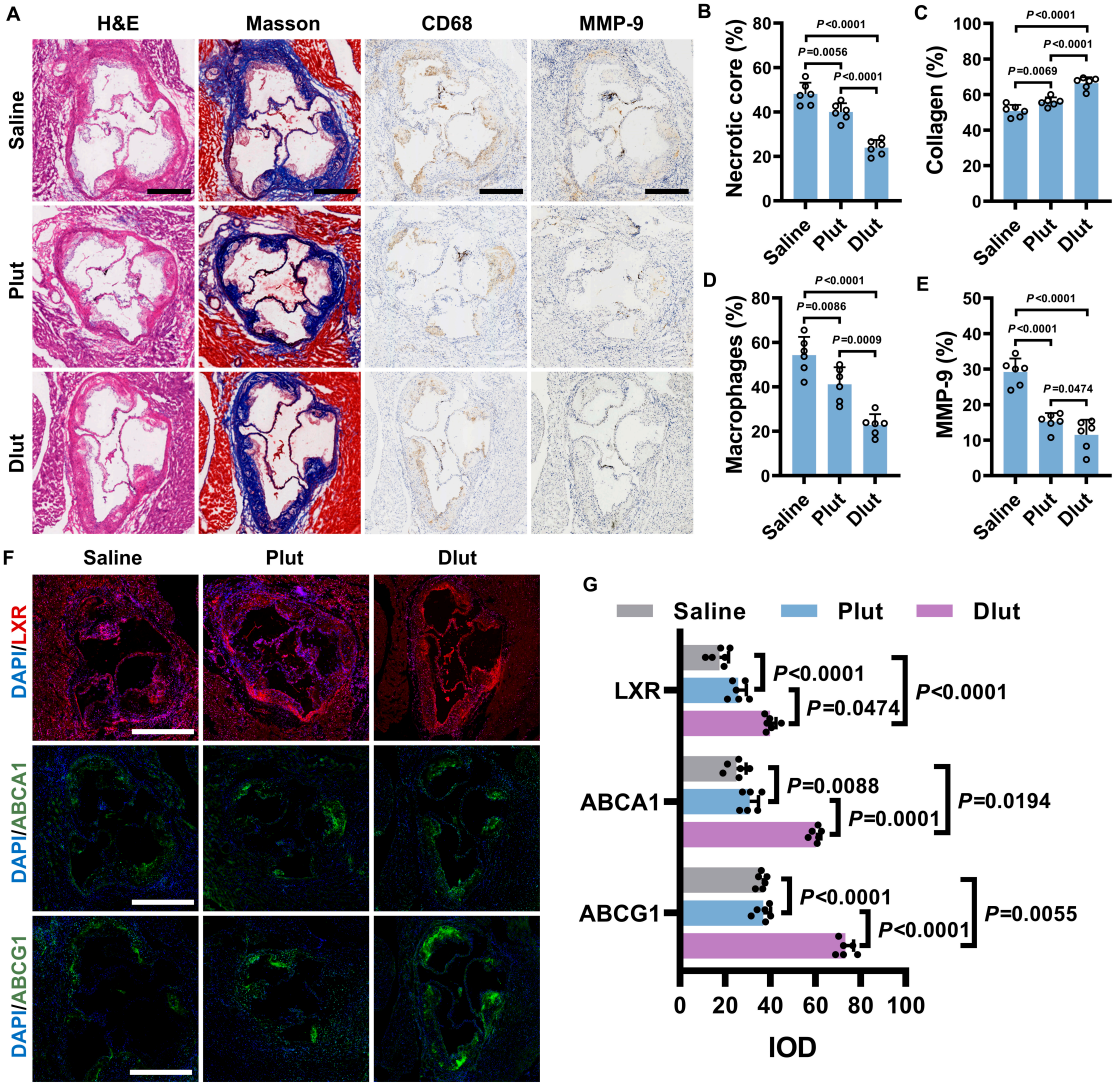
In vivo mechanism explorations of Dlut on atherosclerotic lesions. (A) Section of aortic roots stained with ORO, H&E, Masson’s trichrome, CD68 antibody, and MMP-9 antibody following treatment with saline, Plut, and Dlut. Scale bars, 500 μm. Quantitative analysis results of necrotic core (B), collagen deposition (C), macrophage infiltration (D), and MMP-9 expression (E). Photograph (F) and corresponding quantitative data (G) of aortic root sections stained with fluorescently labeled antibodies to LXR, ABCA1, and ABCG1 after different treatments. Scale bars, 500 μm. Data in (B) to (E) and (G) are expressed as mean ± SD (*n* = 6).

Additionally, hematoxylin and eosin (H&E) staining revealed distinct differences in plaque morphology across treatment groups. The saline-treated group displayed extensive necrotic cores, whereas Plut treatment significantly reduced necrotic areas. Notably, Dlut-treated plaques exhibited structurally compact morphology, indicating a superior ability to suppress pathological cellular necrosis within plaques (Fig. 6A and B). Furthermore, collagen content within the atherosclerotic plaques was determined by Masson’s trichrome staining. As shown in Fig. 6C, the saline-treated group exhibited reduced collagen deposition, consistent with the degradation of the extracellular matrix in vulnerable plaques. In contrast, both Plut and Dlut could protect the collagen structure to some extent, with Dlut demonstrating enhanced efficacy in preventing collagen degradation. Macrophage infiltration and secretion of matrix metalloproteinase-9 (MMP-9) are known to exacerbate plaque erosion [51]. To evaluate these pathological changes, immunohistochemical staining targeting for CD68 (macrophage marker) and MMP-9 was performed (Fig. 6D and E). Compared to the control group, the Plut-treated group exhibited reduced CD68^+^ (macrophage) and MMP-9^+^ immunoreactive atherosclerosis, whereas the Dlut-treated group demonstrated the lowest macrophage infiltration and lowest MMP-9 expression among all cohorts. Furthermore, the influence of Dlut on the macrophages in plaques was detailly studied (Fig. S12). The infiltrated macrophages (CCR2^+^) were inhibited after being treated with Dlut, which indicated a reduced inflammatory environment. On the other hand, Dlut could also enhance the activity of resident-like macrophages (LYVE1^+^), suggesting a microenvironment with balanced immunity.

In sum, by leveraging the active foam cell targeting properties of Lut, Dlut demonstrated superior efficacy in mitigating vulnerable plaque evidenced by reduced lipid deposition, decreased necrosis, diminished macrophage infiltration, and inhibited MMP activity.

Finally, the underlying mechanisms of lipid regulation were further investigated in atherosclerotic mice treated with Plut or Dlut, following the experimental protocol. As illustrated in Fig. 6F, aortic root sections were immunostained with antibodies targeting LXR, ABCA1, and ABCG1. Both Plut and Dlut treatment groups exhibited marked activation of LXR, ABCA1, and ABCG1 expression compared to controls. Notably, Dlut treatment demonstrated superior inhibitory effects compared to Plut, likely due to its enhanced tissue accumulation mediated via active targeting capabilities (Fig. 6G). The selective foam cell targeting strategy of Lut exhibited therapeutic potential in the theranostics of atherosclerosis. The promising therapeutic efficacy of Dlut underscores the clinical potential as a nanoplatform-based therapy.

## Discussion

Despite the promising therapeutic potential of Lut in atherosclerosis, its clinical application was seriously restricted by uncontrollable systematic problems [52–58]. Although nanocarriers enable more efficient drug delivery and reduce toxicity, the additional components required to prepare nanomedicines have also raised concerns about the risk of dose dependency. Therefore, the carrier-free nanomedicine has been developed to overcome this problem. Further, a carrier-free nanomedicine was also endowed with the ability to respond to stimuli in a specific microenvironment, which is considered a traceless release system. Nanomedicines with traceless release properties can be broken down into effective drugs without the need for additional molecules, thus reducing the risk of uncertainty associated with additional components. In the current study, we constructed a Dlut nanomedicine with an active CD44-targeting strategy and stimulus-driven, traceless release of Lut. Dlut demonstrated a robust target efficiency toward atherosclerotic lesions, enabling site-specific accumulation. Moreover, in vivo results indicated that the active targeting strategy outperformed a nontargeting nanomedicine (Plut). On the other hand, its responsiveness to the oxidative stress and acidic microenvironment within plaques helped timely release of Lut in a traceless way. RNA sequencing-based mechanistic investigation revealed that Dlut treatment significantly modulated genes involved in cholesterol metabolism. Three key genes (LXR, ABCA1, and ABCG1) were activated under the treatment of Dlut, indicating that activated lipid efflux might be a central mechanism underlying Dlut’s inhibitory effect on foam cell formation.

Although emerging evidence revealed the therapeutic application of Lut for atherosclerosis, most studies focused on the anti-inflammatory effects [17,59,60]. In this study, we discovered that the Dlut nanomedicine mainly inhibited foam cell formation by regulating lipid metabolism. First, 2 genes that are closely related to cholesterol metabolism (ABCA1 and ABCG1) were found to be down-regulated after oxLDL treatment and reversely up-regulated by Dlut. ABCA1 and ABCG1 are crucial transporters involved in the process of lipid efflux [61,62]. Further, previous studies reported that the transcriptions of both ABCA1 and ABCG1 were regulated by LXR [63,64]. Therefore, we also confirmed the up-regulation of LXR after Dlut treatment both in vitro and in vivo. Our study uncovered the novel cholesterol regulatory mechanism of Lut in foam cells derived from dual lineages, which expanded the possible therapeutic mechanism of Lut.

In conclusion, the carrier-free Dlut nanomedicine improved the in vivo solubility and biosafety of Lut by enabling targeted delivery to the atherosclerotic plaques by recognizing overexpressed CD44. The oxidative stress and acidic microenvironment within plaques triggered the complete disintegration of Dlut as well as the traceless release of Lut. This targeted release inhibited the progression of atherosclerosis by enhancing the lipid reflux via the LXR-ABCA1/G1 pathway. Overall, the therapeutic strategy based on Dlut nanomedicine represents a promising candidate for the targeted treatment of atherosclerosis.

## Materials and Methods

### Preparation and characterization of nanomedicines

Lut (5 mg) and 4-(aminomethyl) phenyl boronic acid (5 mg) were dissolved in a 7-ml solution of DMSO and water (1:1, v/v). After 15 min of stirring, 2 μl of formaldehyde solution was added, followed by the addition of oxDEX solution [10 mg in 0.5 ml of phosphate-buffered saline (PBS)]. After another 4 h of stirring, the solution was added dropwise into PBS solution and dialyzed after 2 h against PBS. Following a similar procedure, the Plut nanomedicine without targeting ability was also prepared as the control (Fig. S13). The NRlut nanomedicine without stimuli responsiveness was prepared with polylysine rather than 4-(aminomethyl) phenyl boronic acid. The particle size of Dlut was confirmed by dynamic light scattering (DLS) as well as the TEM, and the chemical bonding in nanomedicine was also characterized with XPS and FTIR.

### Intracellular uptake of nanoparticles

RAW 264.7 cells or MOVAS cells were seeded in the confocal dishes (10^5^ cells per dish) and stimulated into foam cells. Next, the solution of (Rho)Dlut (1 mg/ml) or (Rho)Plut (1 mg/ml) was added. After being cocultured with (Rho)Dlut/(Rho)Plut for 2, 4, and 6 h, the cells were washed with PBS, fixed with 4% paraformaldehyde, and then stained with 4′,6-diamidino-2-phenylindole (DAPI) for 10 min. On the other hand, the extra HA with various concentrations were added along with (Rho)Dlut. After 4 h of incubation, the fluorescence of (Rho)Dlut in foam cells was observed by confocal laser scanning microscope (CLSM). Images were captured using a Nikon A1 Confocal Microscope (Nikon, Japan), and fluorescence intensity was quantified using ImageJ.

### Foam cell induction and ORO staining

To assess the lipid removal capacity of Dlut, RAW 264.7 cells were initially cultured in 12-well plates at a density of 10^5^ cells per well for 24 h. Before being stimulated into foam cells with LPS (500 ng/ml) and oxLDL (50 μg/ml) (Yiyuan, China) for 24 h, the cells were treated for an additional 4 h with saline, Lut (5 mg/ml), Dlut (1 mg/ml), NRlut (1 mg/ml), dexamethasone (1 nM), or atorvastatin (3 μM). Concurrently, MOVAS cells were cultured in 12-well plates at a density of 5 × 10^4^ cells per well and stimulated into foam cells with oxLDL (80 μg/ml). Cells were washed with PBS and fixed with 4% paraformaldehyde for 20 min, washed with PBS again, and stained with Oil Red O Stain Kit (Nanjing JianCheng, China). Images were acquired using a fluorescent inverted microscope (Olympus, Japan), and ORO-positive area quantitative analysis was performed using ImageJ.

### Immunofluorescence staining

RAW 264.7 cells were initially cultured in 12-well plates at a density of 10^5^ cells per well for 24 h. Before being stimulated into foam cells with LPS (500 ng/ml) and oxLDL (50 μg/ml) (Yiyuan, China) for 24 h, the cells were treated for an additional 4 h with saline, Lut (5 mg/ml), and Dlut (1 mg/ml). Concurrently, MOVAS cells were cultured in 12-well plates at a density of 5 × 10^4^ cells per well and stimulated into foam cells with oxLDL (80 μg/ml). Cells were washed with PBS and fixed with 4% paraformaldehyde for 20 min. The membranes were blocked with 5% bovine serum albumin (BSA) in PBS for 1 h at room temperature and subsequently incubated overnight at 4 °C with specific primary antibodies, such as ABCA1 (Novus, USA) and ABCG1 (Novus, USA). Following washes with PBS, the membranes were incubated with fluorescent secondary antibody and then stained with DAPI for 10 min. Images were captured using a Nikon A1 Confocal Microscope (Nikon, Japan), and fluorescence intensity was quantified using ImageJ.

### Cellular lipid efflux assay

To assess the cellular lipid exclusion capability of Dlut, a mouse oxLDL enzyme-linked immunosorbent assay (ELISA) kit (Elabscience, China) and Dil-oxLDL (Yiyuan, China) were used. After inducing cells to form foam cells as described, the culture medium was replaced with a normal medium for 24 or 48 h, and the supernatant was collected for ELISA analysis. Next, cells were incubated with Dil-oxLDL for 4 h, followed by replacement with a normal medium for an additional 12 h. Images were captured using a Nikon A1 Confocal Microscope (Nikon, Japan), and fluorescence intensity was quantified using ImageJ software.

### Ex vivo imaging on distribution of nanomedicines

As mentioned previously, 16-week-old male ApoE^−/−^ mice were fed with high-fat diet (HFD) for 8 weeks and received intraperitoneal injections of either (Rho)Dlut or (Rho)Plut (100 mg/kg). The mice were sacrificed at 6, 12, and 24 h, and we collected the aorta, heart, liver, spleen, lungs, and kidneys. The fluorescent imaging was receipted by IVIS Lumina LT (Perkin Elmer, USA). Subsequently, the remaining viscera were fixed for H&E staining, and the aortic arch was sliced into sections with plaque. Images were captured using a Nikon A1 Confocal Microscope (Nikon, Japan), and fluorescence intensity was quantified using ImageJ.

### In vivo treatment against atherosclerosis

HFD was performed on 8-week-old male ApoE^−/−^ mice for another 8 weeks to induce the atherosclerosis, while 6 mice were sacrificed stochastically with the aortas separated, followed by longitudinally opening and ORO staining, and sections of the aortic roots were also stained with ORO, which were regarded as the baseline. Subsequently, the remaining mice were randomly assigned to 3 groups (*n* = 6), receiving intraperitoneal injections of either Dlut or Plut (100 mg/kg) every other day for an additional 4 weeks while continuing on the HFD. At the ending point, the mice were euthanized, and their aortas were isolated and stained with ORO, and sections of the aortic roots were stained with ORO, H&E, and Masson. Immunohistochemistry and immunofluorescence were performed on aortic roots to assess CD68, 𝛼-smooth muscle actin (𝛼-SMA), MMP-9, ABCA1, ABCG1, LXR, CCR2, and LYVE1. The area ratio of stain-positive tissue to the plaque was quantified.

### Statistical analysis

All data are presented as mean ± SD. The sample size was not predetermined by statistical methods but was determined based on experimental feasibility and sample availability. Samples were processed in a randomized order. For comparisons between the 2 groups, a 2-tailed unpaired *t* test was employed. For comparisons involving multiple groups, one-way analysis of variance (ANOVA) followed by Tukey’s or Dunnett’s multiple comparison test was performed using GraphPad Prism 8. A *P* value of <0.05 was considered statistically significant.

## Data Availability

The relevant data are available from the corresponding authors upon reasonable request.

## Supplementary Materials

Supplementary 1 Materials and Methods Figs. S1 to S13 References [65–69]
