# Supplementary material for: Luteolin Nanomedicine with Stimulus-Driven Traceless Release for Targeting Treatment of Atherosclerosis by Enhancing Lipid Efflux
Source: Research (Wash D C). 2025 Jul 11;8:0754. doi: 10.34133/research.0754 (PMC12246543; doi:10.34133/research.0754)
Supplement: Supplementary 1 — Materials and Methods Figs. S1 to S13 References [65–69] [file research.0754.f1.docx]

# Supporting Information for

**Luteolin Nanomedicine with Stimuli-Driven Traceless Release for Targeting Treatment of Atherosclerosis by Enhancing Lipid Efflux**

Songzan Chen^†,a,b,c^, Zhaojing Wang^†,a,b,c^, Zhida Shen^†,a,b,c^, Di He^a,b,c^, Lijuan Liu^d^, Lingbo Qian^d,*^, Boxuan Ma^a,b,c*^, He Huang^a,b,c*^

^a^Department of Cardiology, Sir Run Run Shaw Hospital, School of Medicine, Zhejiang University, Hangzhou, China.

^b^Zhejiang Key Laboratory of Cardiovascular Intervention and Precision Medicine, Hangzhou, China

^c^Engineering Research Center for Cardiovascular Innovative Devices of Zhejiang Province, Hangzhou, China

^d^School of Basic Medical Sciences & Forensic Medicine, Hangzhou Medical College, Hangzhou, China.

^†^These authors contributed equally to this work

# Materials and methods S1

## Materials

Luteolin, and 4-(aminomethyl)phenyl boronic acid were purchased from Aladdin. Rhodamine-NHS was obtained from Sigma-Aldrich. Dimethylsulfoxide (DMSO), formaldehyde solution, and all other solvents were purchased from Rhawn Chemistry (Shanghai, China) and used without further purification. Dextran oxide (oxDEX) and benzaldehyde modified polyethylene glycol (PEG-CHO) were purchased according to our previous work^65^.

**Stimuli-Responsive of Nanomedicine**

The Dlut nanomedicine was treated in solution with different concentrations of H_2_O_2_ and various pH, respectively. The variation trend of size was recorded by DLS and the morphology was observed by TEM. Furthermore, the drug release behavior of Dlut was evaluated under the trigger of ROS (0.1 mM H_2_O_2_) and acid (pH 6.5). Briefly, a dialysis bag (Spectrumlabs, MWCO = 3500) was filled with nanomedicine solution (2 mL) and incubated with or without the addition of 1 mM H_2_O_2_ at pH 6.5 or 7.4. The samples outside the bag (1 mL) were withdrawn at selected time intervals while 1 mL fresh release medium was added. The released Lut was calculated with HPLC.

## Characterization

The particle size of nanoparticles was recorded by dynamic light scattering (DLS) on a Malvern Zetasizer Nano ZS. The morphology was observed by a Hitachi H-600 transmission electron microscope (TEM) with an accelerating voltage of 10 KV and the sample was dyed with uranyl acetate before measurement. Fourier transform infrared spectra of the freeze-dried nanoparticles were obtained with ATR-FTIR (Spectrum One, Nicolet). XPS measurement was performed with an XSAM 800 X-ray photoelectron microscopy (Kratos, UK).

## Western Blot

RAW 264.7 cells were initially cultured in 12-well plates at a density of 10^5^ cells per well for 24 hours. Before being stimulated into foam cells with LPS (500 ng/mL) and oxLDL (50 μg/mL) (Yiyuan, China) for 24 hours, the cells were treated for an additional 4 hours with saline, luteolin (Lut, 5 mg/mL), Dlut (1 mg/mL), GSK2033 (20 μM) or DIDS (200 μM). Concurrently, MOVAS cells were cultured in 12-well plates at a density of 5 x 10^4^ cells per well and stimulated into foam cells with oxLDL (80 μg/mL). Cells were harvested as described and lysed using RIPA buffer with PMSF. Protein concentration was determined using the BCA assay kit (Beyotime, China). The proteins were then resolved via SDS-PAGE and transferred onto a PVDF membrane (Millipore, USA). The membranes were blocked with 5% skimmed milk for 1 hour at room temperature and subsequently incubated overnight at 4 °C with specific primary antibodies, such as ABCA1(Novus, USA), ABCG1(Novus, USA), LXR (Abcam, UK) and PPARγ (Abcam, UK). Following washes with TBST, the membranes were incubated with secondary antibodies. Protein bands were visualized using the VILBER ECL Western Blotting Substrate and quantitatively analyzed using ImageJ software.

## RT-qPCR Assay

Total RNA was extracted from cells using the RNA-Quick Purification Kit (ES Science, China). Subsequently, the RNA was reverse transcribed into cDNA using the Evo M-MLV RT Premix (Accurate, China) for qPCR. Quantitative PCR was performed using the Hieff® qPCR SYBR® Green Master Mix (Yeasen, China) with gene-specific primers on the QuantStudio 6 Real-Time PCR system (Thermo Fisher Scientific, USA). β-actin was employed as the housekeeping gene. The relative changes in gene expression were analyzed using the 2^−ΔΔCT^ method based on the RT-qPCR results.

**Flow Cytometry**

RAW 264.7 cells were initially cultured in 12-well plates at a density of 10^6^ cells per well for 2 hours, after which the medium was replaced with a basal medium to induce macrophage polarization. M1-type polarization was achieved using LPS(500 ng/mL), while M2-type polarization was induced with IL-4 (50 ng/mL) and IL-13 (50 ng/mL). Subsequently, the cells were harvested and stained with CD80, CD206, or CD44 antibodies (Invitrogen, USA). The stained cells were then analyzed using a fluorescence-activated cell sorter (Beckman Coulter, USA).

## *In vivo* acute toxicity evaluation

ApoE^−/−^ mice were treated with saline and Dlut (100 mg kg^-1^). The mice were sacrificed after 24 h, and the major organs (heart, liver, spleen, lung, and kidney) were isolated. The histological sections were prepared and stained with hematoxylin and eosin (H&E).

## *In vivo* pharmacokinetics study

ApoE^-/-^ mice were administrated with (Rho)Dlut (10 mg kg^-1^). At predefined time points, an equal volume of whole blood samples was collected. The mean fluorescence intensity was determined to calculate the metabolism of nanomedicine.

## RNA‑seq and data analysis

RNA of the 3 groups described above was extracted by Trizol following the manufacturer’s protocols (Invitrogen, US). Each group includes 3 biological replicates. After primary qualification, eligible RNA was sent to Hangzhou Cosmos Wisdom Biotech Co., Ltd for cDNA library construction. Next, the library was sequenced on the Illumina Hiseq 4000 platform. Next, the raw reads were further processed on Cosmos Wisdom cloud platform. Afterward, raw data were cleaned of low-quality reads and sequences with adapters. Finally, clean reads were used for subsequent analysis and aligned to the NCBI mouse reference genome for subsequent analysis. The raw RNA count matrix was normalized using DESeq2 using variance stabilizing transformation (vst). The genes with abs *log2 fold-change (FC)* ≥ 1 and *p-value <* 0.05 were regarded as DEGs. PCAs comparing gene FPKM values were performed using the prcomp function in R (version 4.0.5). PCA excluded genes that with low expression across all samples (maximum FPKM value ≤ 10). PCA plots were visualized using the R package ggbiplot (version 0.6.2).

To evaluate the gene distribution among the three groups, the triwise plots were performed (version 0.99.5)^66^. To better visualize the regulatory pattern of Dlut under oxLDL environment, the genes upregulated by oxLDL while further downregulated by Dlut were shown in red dots. On the other hand, the genes downregulated by oxLDL while further reversed by Dlut were shown in blue dots.

To further explore the genes regulated by Dlut under oxLDL environment, we focused on the genes that were significantly changed by oxLDL but reversed by Dlut (abs *log2 FC* ≥ 0.5 and *p-value <* 0.05). These genes were further enriched for the related diseases and KEGG pathways. The DOSE package (v3.16.0) was used to conclude the disease ontology according to the DisGenet database ^68, 67^. Meanwhile, the ClusterProfiler (v4.10.0) package was selected to perform the KEGG pathway enrichment analysis based on the identified DEGs^69^. The gene-disease network was further visualized by the enrichplot package (version 1.10.2)^70^.


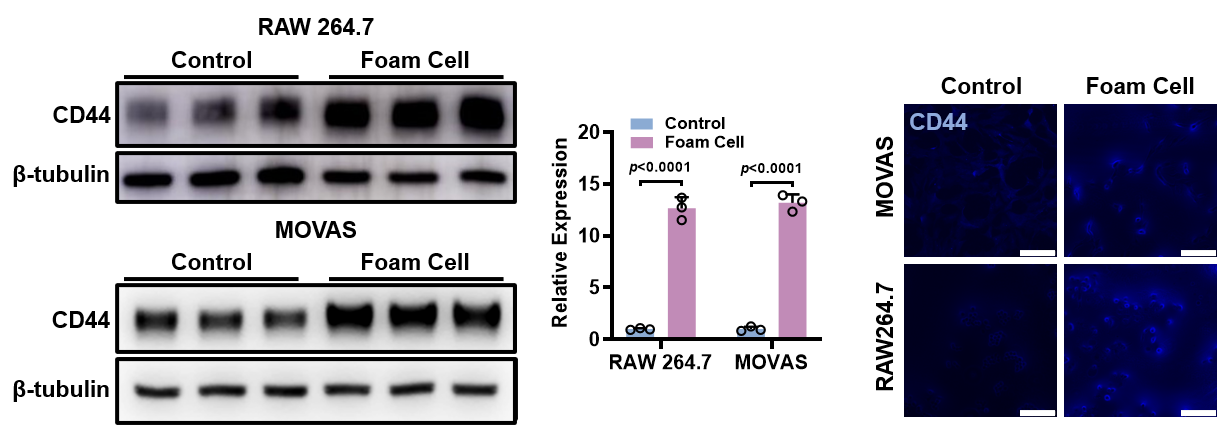


**Figure S1.** Relative expression of CD44 in RAW 264.7 and MOVAS after activation of LPS/oxLDL. The upregulation of CD44 protein in RAW 264.7 (upper panel) and MOVAS (lower panel) was verified. Corresponding quantitative data of CD44 during foam cell formation was illustrated. The fluorescence intensity of CD44 protein expression within MOVAS and RAW264.7 was measured during foam cell formation. Data are expressed as mean ± s.d. (n = 3). t.test was used to calculate p values.


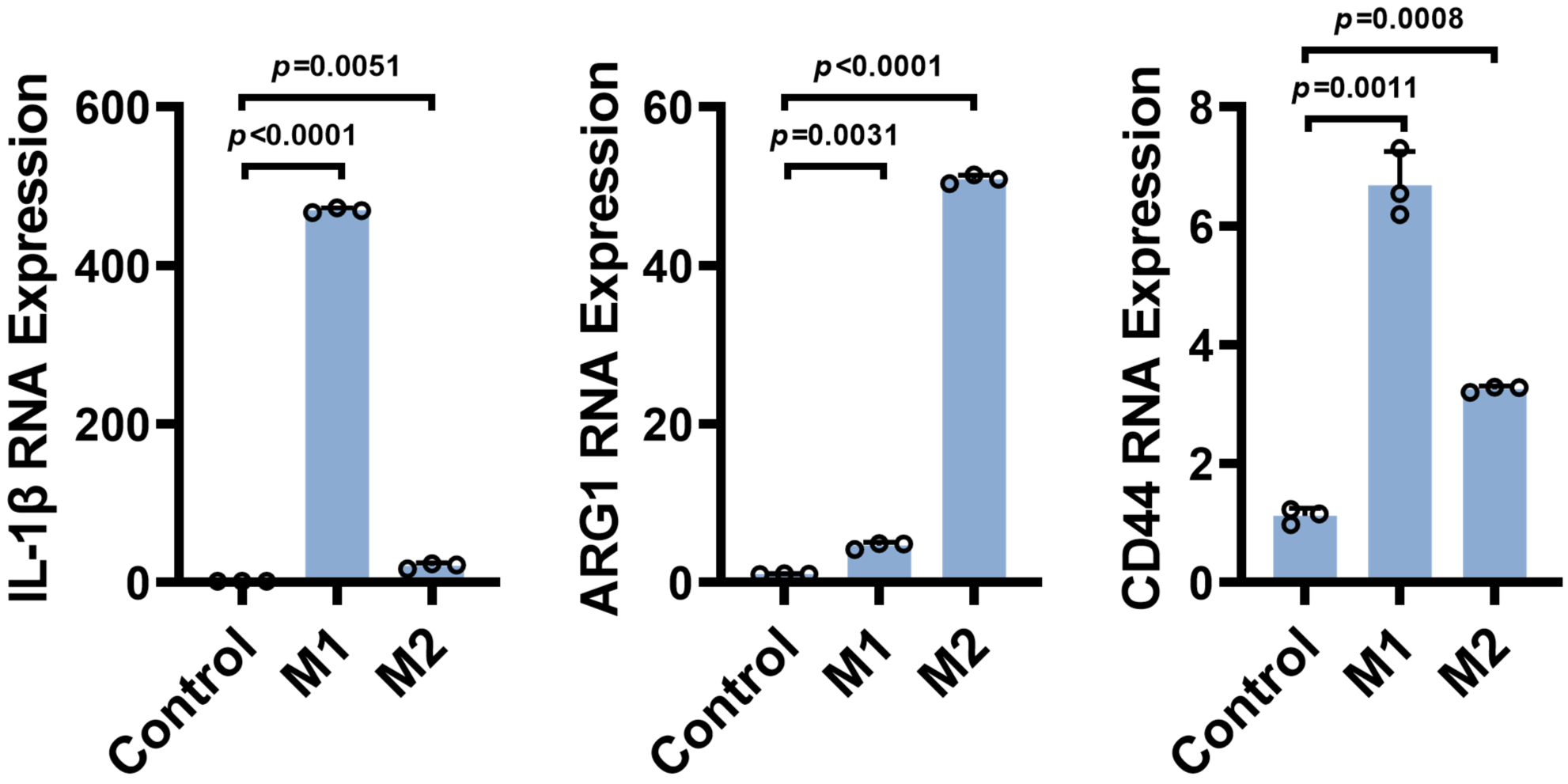


**Figure S2.** The mRNA changes of CD44 during macrophage polarization. The polarization of macrophages was confirmed by the classical M1 (IL-1β) and M2 (ARG1) markers. The significant upregulation of CD44 was identified during macrophage M1 polarization. Data are expressed as mean ± s.d. (n = 3). t.test was used to calculate p values.


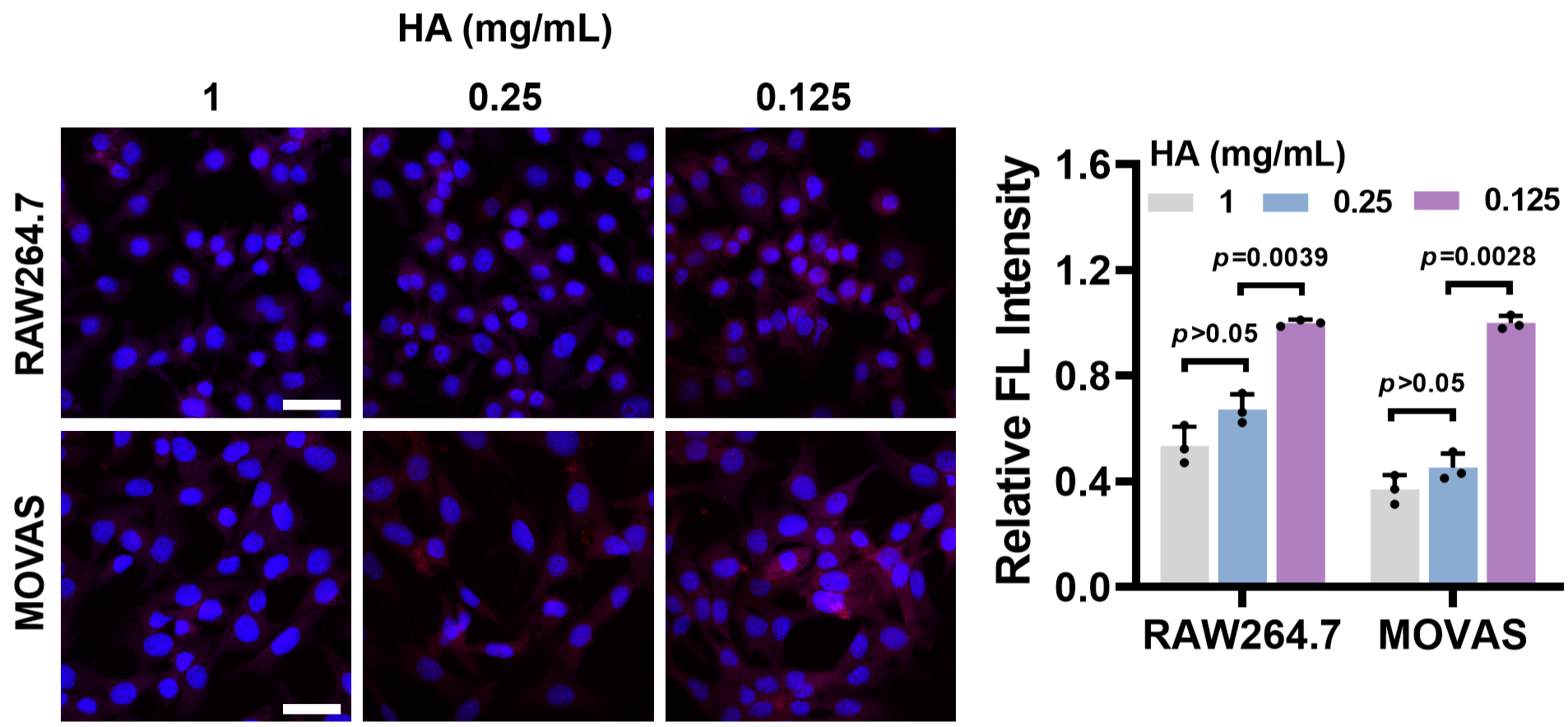


**Figure S3.** Fluorescent photographs and quantitative intensity of Dlut internalization in foam cells after 4 h with different concentrations of HA. Data are expressed as mean ± s.d. (n = 3). t.test was used to calculate p values.


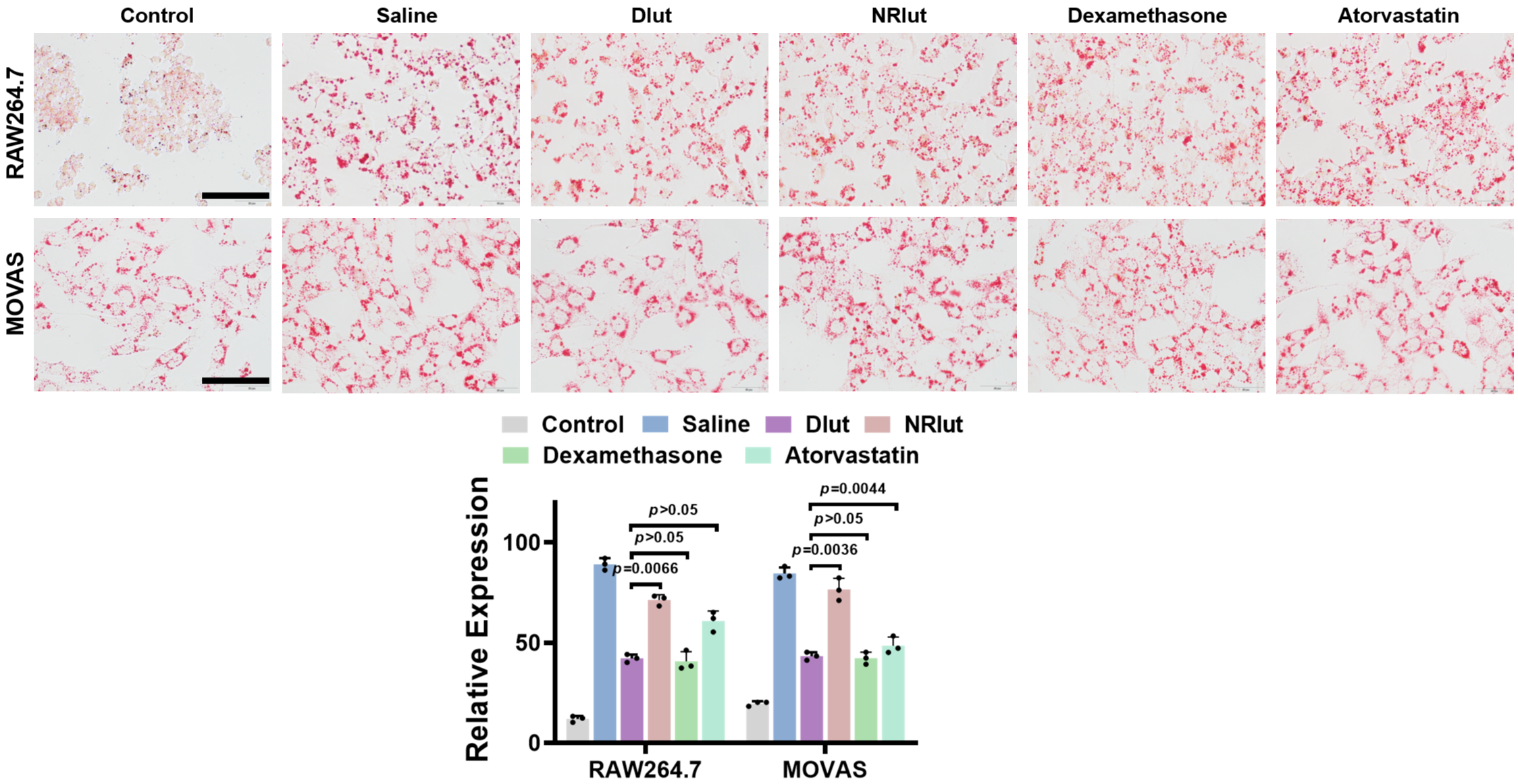


**Figure S4.** ORO staining and the quantification data of foam cells in vitro with different stimulations. A control group without stimuli-responsiveness (NRlut) was used as a negative control while the classical anti-inflammatory drug (Dexamethasone) and the anti-atherosclerosis drug (Atorvastatin) were selected for the positive control. Data are expressed as mean ± s.d. (n = 3). t.test was used to calculate p values.


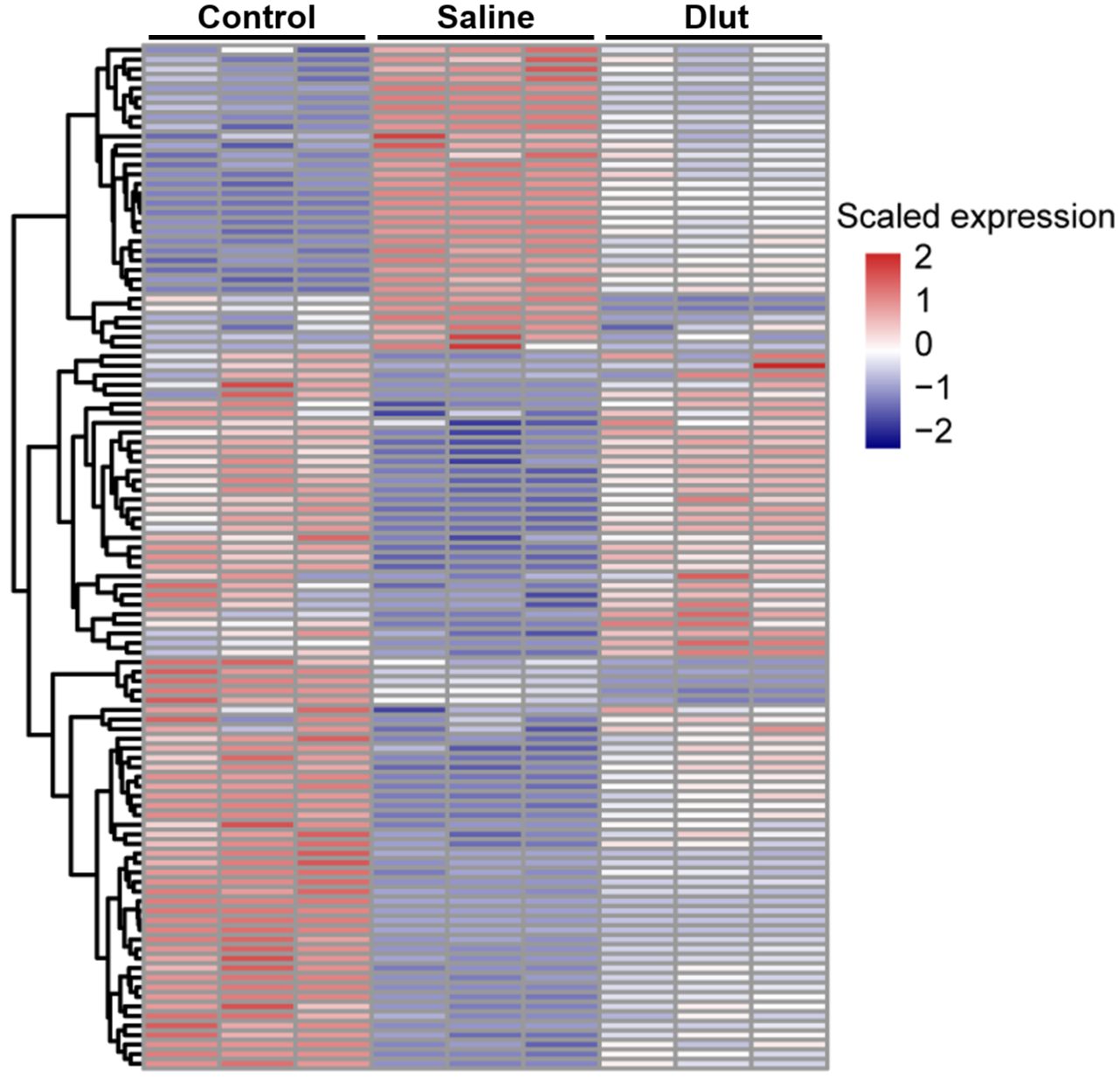


**Figure S5.** Heatmap of differentially expressed genes in foam cells after various treatments.


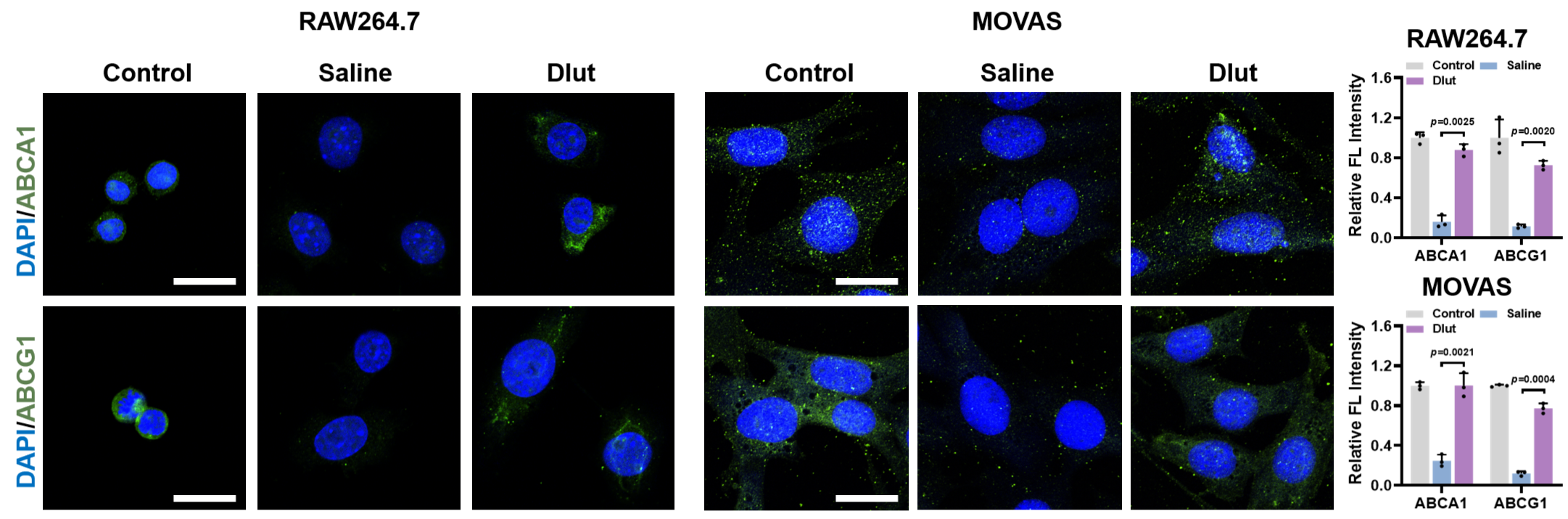


**Figure S6.** Photograph and quantification of ABCA1/G1 protein expressions within foam cells by immunofluorescent staining. Data are expressed as mean ± s.d. (n = 3). t.test was used to calculate p values.


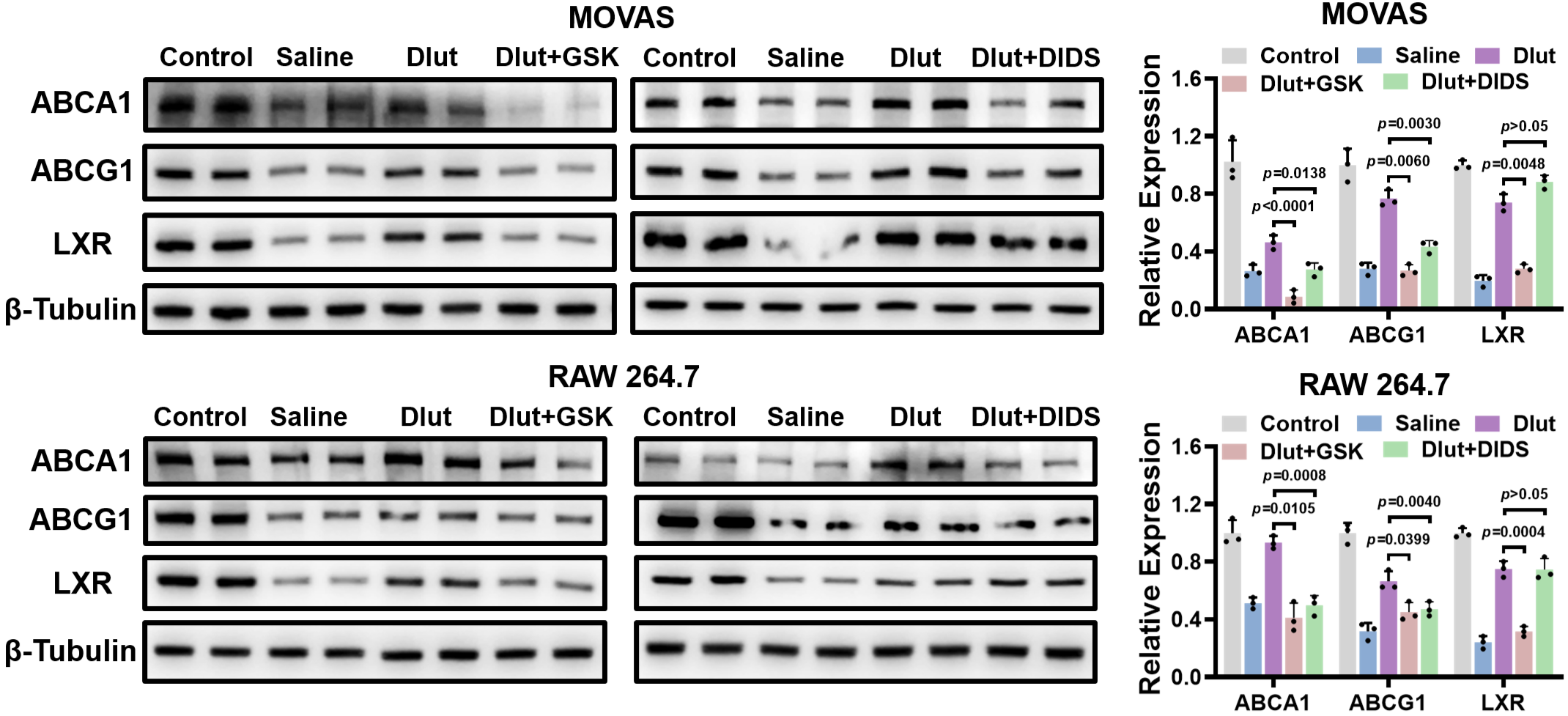


**Figure S7.** The protein expressions and quantitative data of the LXR-ABCA1/G1 pathway in foam cells derived from MOVAS and RAW264.7 were treated with Dlut along with LXR inhibitor (GSK2033) and ABC inhibitor (DIDS). Data are expressed as mean ± s.d. (n = 3). t.test was used to calculate p values.


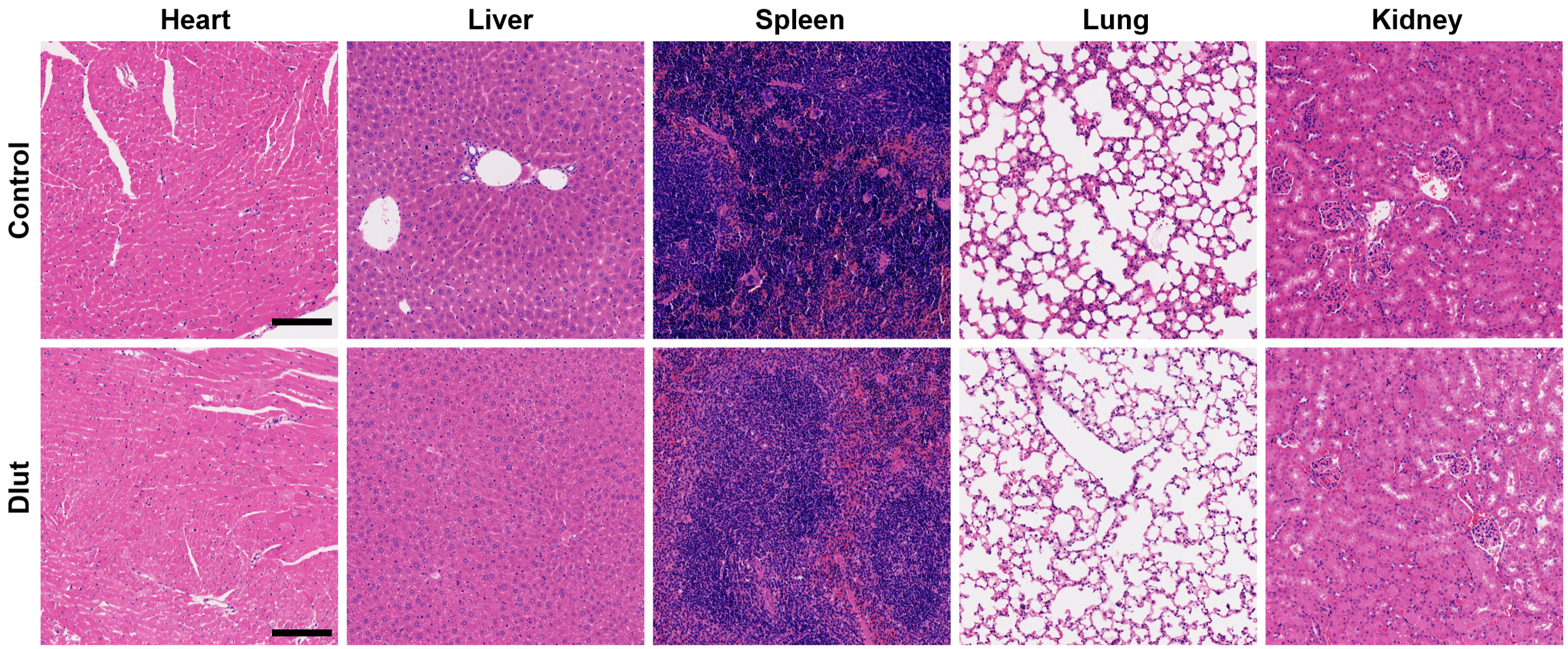


**Figure S8.** H&E stained sections of major organs from mice treated with saline and LAID. The scale bars were 100 μm.


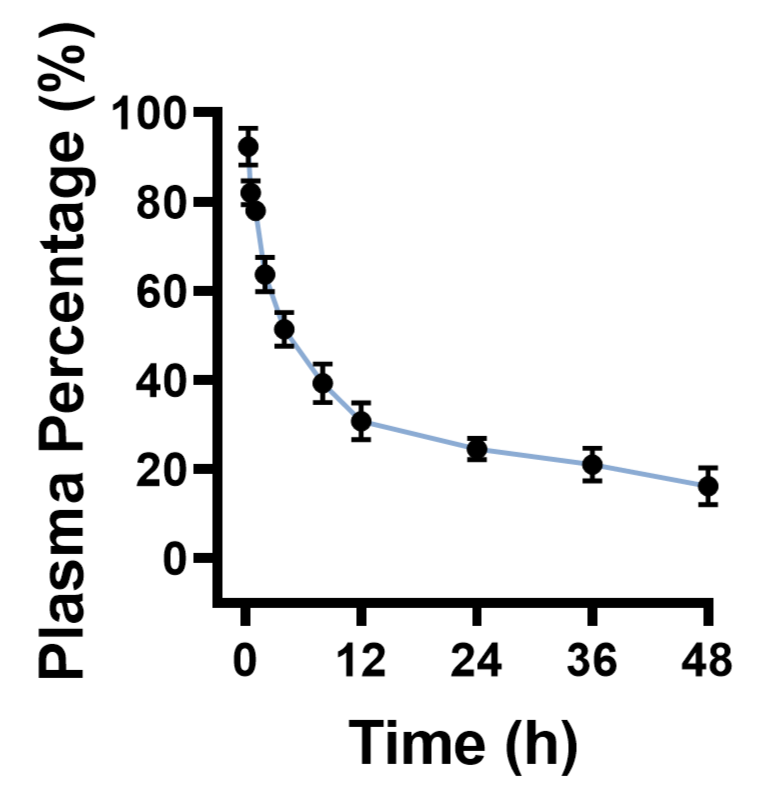


**Figure S9.** The pharmacokinetics of Dlut in blood in 48 h. Data are expressed as mean ± s.d. (n = 3).


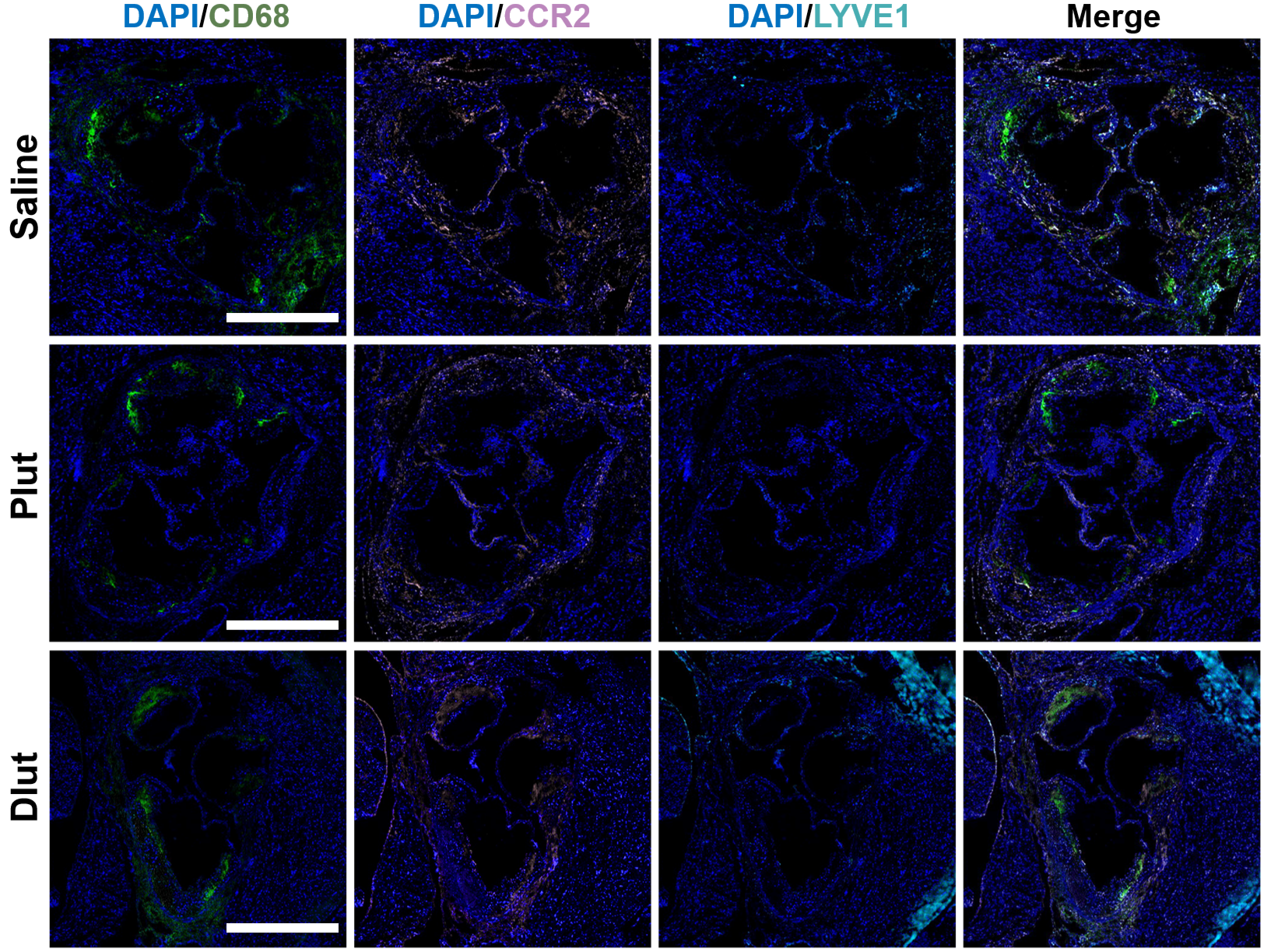


**Figure S10.** The treatment of Dlut inhibited the accumulation of recruited macrophages (CD68^+^CCR2^+^) while enhancing the activities of resident-like macrophages (CD68^+^LYVE1^+^) in lesions.


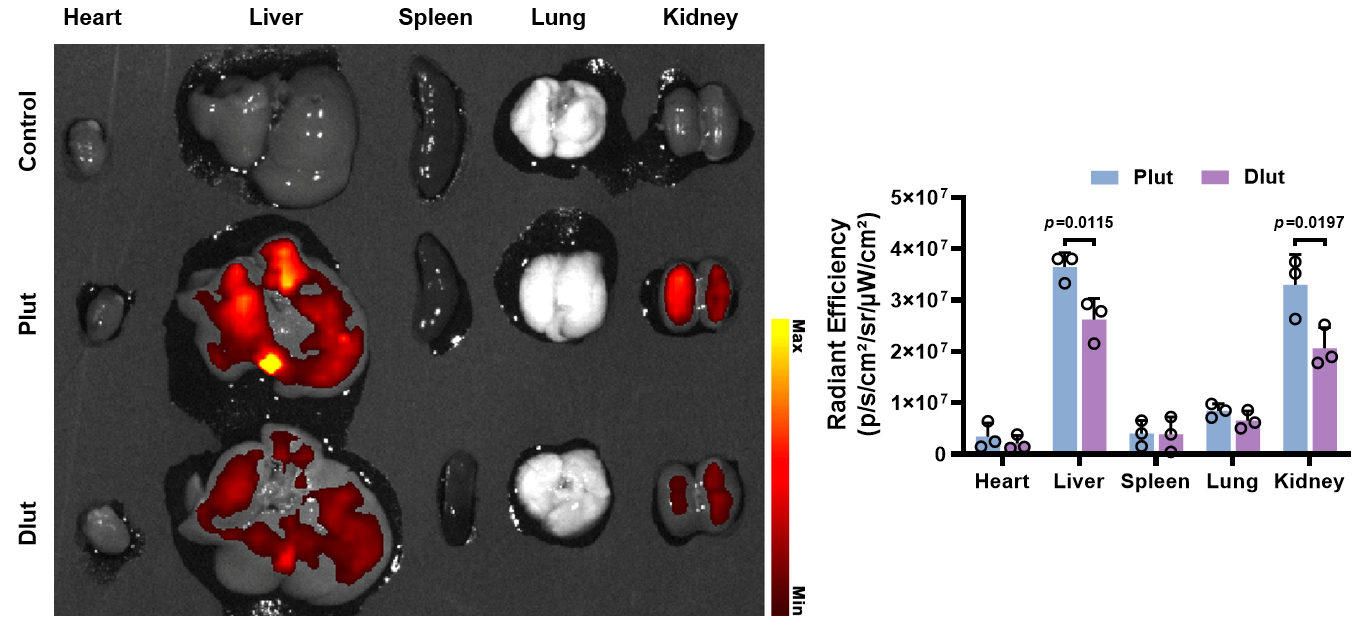


**Figure S11.** *Ex vivo* images and fluorescent intensity data of mean organs of mice treated with Plut and Dlut. Data are expressed as mean ± s.d. (n = 3). t.test was used to calculate p values.


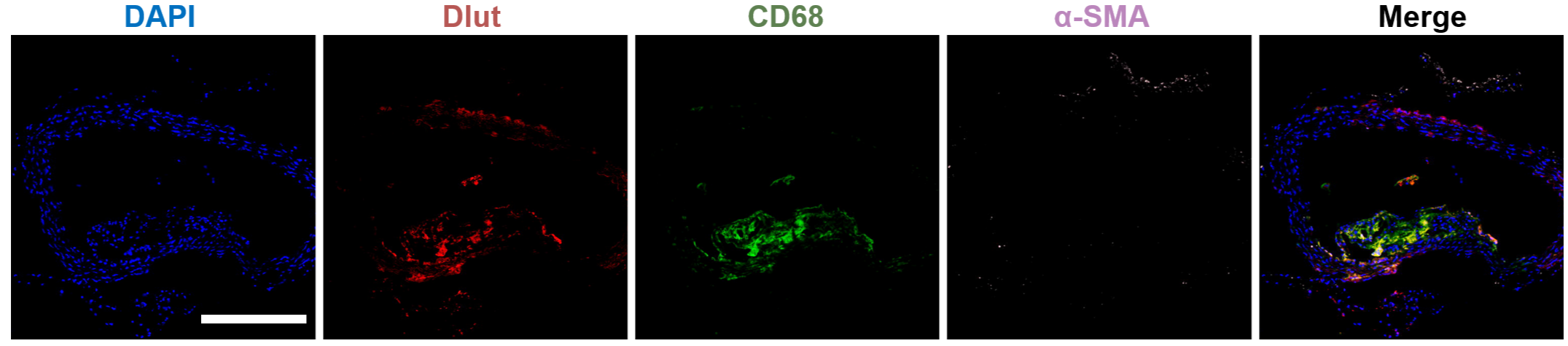


**Figure S12.** IF confirmed the co-localization of Dlut with macrophages (CD68^+^) and SMCs (α-SMA) within plaques.


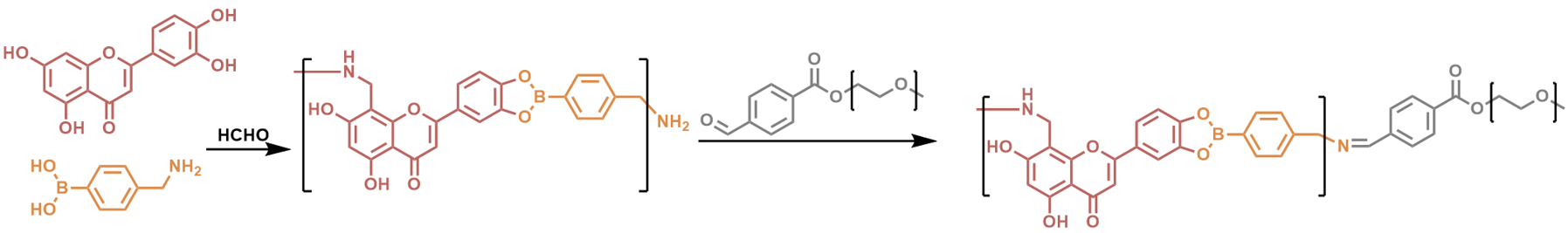


**Figure S13.** Synthetic route of Plut nanomedicine.
